# Does intermediate care improve patient outcomes or reduce costs?

**DOI:** 10.1186/s13054-015-0813-0

**Published:** 2015-03-02

**Authors:** Jean-Louis Vincent, Gordon D Rubenfeld

**Affiliations:** Department of Intensive Care, Erasme Hospital, Université libre de Bruxelles, Brussels, 1070 Belgium; Program in Trauma, Emergency, and Critical Care, Sunnybrook Health Sciences Center, Interdepartmental Division of Critical Care, University of Toronto, Toronto, ON M4N 3 M5 Canada

## Abstract

ICUs are an essential but expensive part of all modern hospitals. With increasingly limited healthcare funding, methods to reduce expenditure without negatively influencing patient outcomes are, therefore, of interest. One possible solution has been the development of ‘intermediate care units’, which provide more intensive monitoring and patient management with higher nurse:patient ratios than the general ward but less than is offered in the ICU. However, although such units have been introduced in many hospitals, there is relatively little published, especially prospective, evidence to support the benefits of this approach on costs or patient outcomes. We review the available data and suggest that, where possible, a larger unit with combined intermediate care and intensive care beds in one location may be preferable in terms of greater flexibility and efficiency.

## Introduction

Intensive care medicine is still a relatively young specialty in comparison with other fields of medicine, but has developed to become an essential part of all modern hospitals and the ICU now takes up a large proportion of the hospital budget. The challenge of caring for low-risk ICU patients effectively and efficiently dates back to the earliest days of organized intensive care [1]. The fundamental question is how best to organize the available human and technological resources to address the needs of patients who do not now, but might soon, need advanced critical care interventions. Healthcare, hospital and ICU managers are increasingly faced with difficult financial decisions regarding how best to allocate increasingly limited funds without reducing standards of care or negatively impacting on patient outcomes. In the search for solutions to this funding dilemma, methods to reduce ICU costs have been proposed. One such approach involves the use of so-called ‘intermediate care’, ‘high-dependency’, ‘step-up/down’ or ‘progressive care’ units. For the sake of this article, we will use the term ‘intermediate care unit’ (IMCU) and assume that all such units are similar [2], although in some hospitals several different types of unit may be used to progressively move a patient from the ICU to the general ward. Some hospitals have created general IMCUs, whereas others have created IMCUs for specific patients groups, such as cardiac, neurosurgical, or respiratory patients. In an international observational study conducted in 2007, 31% of the participating hospitals in 75 countries had an IMCU [3].

The general concept is that an IMCU can be used to manage patients who need more care than a general ward can provide but do not really need the degree of monitoring and expertise that an ICU offers; such units can, therefore, theoretically be run with lower nurse:patient ratios and less equipment than ICUs and hence are often seen to be a cheaper alternative. Do these units really reduce costs and do patients benefit from this stepwise approach? Although the different types of IMCU and lack of standardized definitions for such units can make it difficult to compare data from different studies, here we will consider the available evidence.

## Intermediate care units: the evidence for and against

It is well recognized that, although numbers of ICU beds are limited, many patients are admitted to the ICU who do not really need full ‘intensive care’ [1]. Proponents of the IMCU suggest that such units could, therefore, be used to free up ICU beds for patients most in need of full ICU facilities and expertise. In one large multicenter study in the US, 20% of all ICU admissions were considered ‘low severity’ and potentially ‘clinically unnecessary or inappropriate’ [4]. Other more recent studies have reported that 20 to 30% of all ICU patients are admitted for less than 24 hours for routine surveillance/monitoring [5,6]; this percentage may be considerably higher on surgical ICUs than on medical or mixed units. Such patients are at low risk of developing complications and hence unlikely to require invasive therapy during their ICU stay [1,4,7,8], making them potentially appropriate candidates for IMCUs with higher staff:patient ratios and more specialized equipment, notably for monitoring, than on the general ward but less than on the ICU, theoretically making these units a more cost-effective option for patients not needing full ICU facilities.

As well as direct admissions to the IMCU for low-risk patients requiring short-term intensive monitoring, these units are also used as a ‘step-down’ facility for patients who no longer need intensive therapy, but are perhaps not ready to return to the general ward [9]. Without the presence of an IMCU, such patients would, perhaps unnecessarily, be kept on the ICU, thus occupying a bed and preventing its use by a patient who may benefit more. Alternatively, they may be discharged to the ward early, especially in small ICUs with limited bed availability, carrying the risk of increased readmission rates, which have been associated with increased mortality rates [10]. IMCUs are also used as ‘step-up’ units, admitting patients who require more intensive management than is available on the general floor but do not yet need full intensive care, in the hope that this move would prevent a later ICU admission.

### Effects on outcomes?

Despite the theoretical reasons to support the use of IMCUs and the many such units now in existence, there are relatively few published data directly assessing their value and the studies that have been published are mostly retrospective in nature. In an early study, Franklin and colleagues [11] reported that the opening of an IMCU was associated with a decrease in mortality rates across the medical service, largely because of a decrease in the numbers of deaths on general medical wards. The authors suggested that this was in part because more ICU beds had been made available as lower-risk intensive monitoring patients were admitted to the IMCU so that high-risk patients who would otherwise have been managed on the general ward because of bed shortages were being more appropriately managed on the ICU. Beck and colleagues [12] reported that patients with high severity scores who were discharged to hospital wards had a higher risk of in-hospital death compared with patients discharged to a high-dependency unit, suggesting that the IMCU helped prevent ‘premature’ discharges to the ward. However, Campbell and colleagues [13] reported that discharge to a high-dependency unit was an independent risk factor for early ICU re-admission, suggesting that these patients had been discharged prematurely; importantly, as mentioned earlier, ICU readmission is associated with increased mortality rates [10]. In a before-after study in a surgical ICU, opening an IMCU was associated with an increase in the overall severity of illness of the patients admitted to the ICU, but without increased mortality [14]. The creation of a step-up ‘subintensive care unit’ within an acute care for the elderly department was associated with improved patient outcomes compared with an historical cohort of patients with similar disease severity admitted to the general acute care ward [15]. However, in a retrospective cohort study of data from 28 ICUs in the Netherlands, the presence of an IMCU was associated with higher in-hospital mortality than if no such unit was available [16]. In a 14-year observational study, Teli and colleagues [17] reported a significant decrease in ICU admissions for routine vascular surgical patients after creation of a high-dependency unit. Finally, Ranzani and colleagues [18] performed a propensity-matched analysis of 160 patients discharged from the ICU to an IMCU over a 5-year period in a teaching hospital in Brazil. Ninety-day mortality rates and unplanned ICU readmissions were similar in patients discharged to the IMCU and those discharged to the general ward.

Few studies have prospectively collected mortality data for comparisons of intermediate and intensive care. In one study, Bellomo and colleagues [19] found that the opening of a four-bed high-dependency unit in their department had no effect on mortality rates or hospital length of stay and was associated with an increase in the number of patients requiring re-intubation. In a prospective before-after study, Solberg and colleagues [20] reported that introduction of an IMCU, for use as a step-down unit from the ICU, was associated with improved ICU utilization and more appropriate use of ICU beds, such that a sicker population of patients, with higher mortality rates, was admitted to the ICU during the IMCU period. However, there were no differences in the numbers of ICU referrals, readmissions to the ICU, or ICU length of stay before and after the IMCU was opened.

In a recent multinational observational study performed in 167 ICUs from 17 European countries, the presence of an IMCU in the hospital was associated with a significantly reduced adjusted hospital mortality for adults admitted to the ICU (odds ratio 0.63, 95% confidence interval 0.45 to 0.88, *P* = 0.007), notably those admitted for full intensive care therapy rather than just for monitoring [21]. However, in this study less than 25% of the ICU patients actually received treatment in an IMCU either before or after their ICU admission. Hence, if IMCUs do reduce mortality, they must do so by somehow improving the performance of the ICU in caring for the sickest patients who never receive care in the IMCU. The mechanism for this effect on care outside the IMCU is not clarified in the study. In addition, the apparent ‘harm’ of not having an IMCU was derived from data on a minority (16%) of the participating ICUs that were smaller and less likely to be academic centers than ICUs in hospitals with IMCUs.

### Effects on costs?

The theoretical increased efficiency associated with availability of an IMCU and the reduced staff:patient ratios on such units have often been promoted as likely to be associated with reduced costs. In an early systematic review of the literature, only three studies were identified that had conducted economic analyses and they all concerned respiratory IMCUs. The authors concluded that there were insufficient data to support ‘the viewpoint that the addition of a [transitional care unit] to an institution with ICU and general ward beds is cost-effective’ [22]. In a more recent prospective study, Bertolini and colleagues [23] reported that, for patients with exacerbation of chronic obstructive pulmonary disease, the total cost per patient was lower in a respiratory IMCU than on an ICU. However, Solberg and colleagues [24] noted that, although the costs of an IMCU day were less than those of an ICU day, total hospital costs per patient increased significantly after introduction of an IMCU. The authors suggested that this was due to a greater severity of illness in the patients admitted after introduction of the IMCU and hence longer ICU stays and was not related to the introduction of the IMCU *per se*. Moreover, they suggested that their IMCU may have been running at less than optimal efficiency, because only 85% of beds were occupied during the study period as a result of staffing constraints. In a Canadian hospital in which the IMCU was closed for budgetary reasons, Byrick and colleagues [25] reported an increase in the numbers of admissions to the ICU with low severity of illness and with short stays and an increase in the numbers of patients discharged with low nurse workload requirements; these findings were interpreted as an increase in inefficient use of staff and resources and the IMCU was reopened.

Assessing cost differences occurring as a result of introducing IMCU beds can be difficult, with the existing literature using the typical bed-day cost algorithm, which does not factor in the reduced acuity and cost of patients as they move through the ICU and hospital, such that an ICU day cost before they move to the ward is very similar to the first day on the ward cost [26]. The cost savings achieved by shifting patients from one bed to another (from an ICU to a long-term acute care facility or an ICU to the ward a few days earlier) need to be calculated as the total cost of care, not just the ICU costs.

## An alternative approach?

Despite the introduction of general IMCUs in many hospitals, particularly in Europe, there are relatively few data evaluating their role in improving patient outcomes and/or reducing costs. Indeed, the few prospective data that are available suggest that such units have little effect on outcomes or costs. This finding is not so surprising. First, although creation of IMCUs may theoretically reduce overcrowding of ICUs, in practice this approach does not solve the problem but merely shifts it to another location. Second, creating additional IMCU beds may have the unintended consequence of creating demand for beds that did not exist previously, with the only outcome being increased cost. There is large variability in the United States for decisions to admit to an ICU even at the lowest end of acuity [27]. When Taiwan created special weaning units and when the United States created long-term acute care hospitals to move chronically ventilated patients out of acute care ICUs, the major effect both countries saw was an ‘epidemic’ of chronic critically ill [28,29]. Third, patients admitted to an IMCU will generally be low-risk and would have consumed a relatively low proportion of total ICU costs if admitted to the ICU [24,30]; these patients are not the ‘big spenders’ of ICU care. In addition, as intermediate care is more costly than general ward care, and as some patients would be managed on the general ward if no IMCU were available, total hospital costs may in fact increase with introduction of such a facility.

An alternative solution may be to combine intermediate care beds with intensive care beds in one location [31]. These combined units may be able to operate more efficiently than separate units. Essential, often expensive monitoring and interventional equipment can be concentrated in the one area rather than having to be duplicated in two separate units. There is also greater flexibility in bed use and staffing on a larger combined unit than in separate units. Sudden fluxes in demand for beds or change in the types of patients being admitted can be managed more easily in a larger unit, and bed designations can be changed from intermediate to intensive care as necessary. The nursing and medical staff also benefit from caring for different types and categories of patients, making working conditions more varied and interesting.

In an analysis of expenditure data from 72 adult intensive care and combined intensive care/high-dependency units in the UK, Jacobs and colleagues [32] reported that larger units, which combined ‘intensive' with ‘intermediate’ beds, may be associated with reduced costs compared to smaller units. Mortality rates in larger, high-volume ICUs may also be lower than in smaller units with fewer annual admission rates [33]. An Expert Group of the UK Department of Health reported in 2000 that ‘the existing division into high dependency and intensive care based on beds be replaced by a classification that focuses on the level of care that individual patients need, regardless of location’ and suggested that wherever possible all critical care beds should be in adjacent locations [34].

An evidence-based approach to this organizational question is difficult. First, it is unlikely that we will ever be able to do a definitive randomized trial evaluating IMCUs. The ideal study evaluating IMCUs would not only focus on ICU costs and outcomes but also on the costs and outcomes of hospital patients who use the IMCU and are never cared for in the ICU. Second, local factors confound comparison of hospital organizational structures. For example, hospital policies regarding ward use of intravenous insulin, non-invasive ventilation, and cardiac telemetry make admission to an ICU bed mandatory in some hospitals and not others [35]. Variation in nursing ratios and the availability of other staff make ward care of selected patients safe in some hospitals but not others. Third, the cost-savings achieved by introducing an IMCU is also unclear. The most expensive part of intensive care is nursing care and assuming the nursing ratios and numbers of patients are fixed, introducing an IMCU will not reduce costs significantly. Therefore, simply shifting ICU days to IMCU days will not necessarily translate into cost-savings. In fact, easier access to these beds might increase the overall number of patient bed days in an intensive care area. Finally, solutions must reflect local realities. While a large single ICU with flexible staffing and monitoring may be ideal for efficiency and quality, some hospitals may simply not have the physical space to create this solution. In these cases, a separate IMCU may be the only way to increase critical care capacity. As in many areas of health services research and quality improvement, one size may not fit all.

## Conclusion

There is little published evidence to support a positive effect of separate IMCUs on efficiency, costs or patient outcomes. However, local considerations, including demand, ICU and ward case-mix, ICU volume, available staffing, physical and financial resources, and the quality of clinical care on the ward, must be taken into account when making such decisions. Importantly, whether or not an IMCU is present, the need for careful patient triage must be maintained to ensure that sufficient ICU beds are available at all times for those patients who will benefit.

## Abbreviation

IMCU: Intermediate care unit

## References

[1] Wagner DP, Knaus WA, Draper EA, Zimmerman JE (1983). Identification of low-risk monitor patients within a medical-surgical intensive care unit. Med Care..

[2] Stacy KM (2011). Progressive care units: different but the same. Crit Care Nurse..

[3] Sakr Y, Moreira CL, Rhodes A, Ferguson ND, Kleinpell R, Pickkers P, et al. The impact of hospital and ICU organizational factors on outcome in critically ill patients: results from the extended prevalence of infection in intensive care study. Crit Care Med. 2014. Epub ahead of print. 10.1097/CCM.0000000000000754 25479111

[4] Rosenthal GE, Sirio CA, Shepardson LB, Harper DL, Rotondi AJ, Cooper GS (1998). Use of intensive care units for patients with low severity of illness. Arch Intern Med..

[5] Vincent JL, Rello J, Marshall J, Silva E, Anzueto A, Martin CD (2009). International study of the prevalence and outcomes of infection in intensive care units. JAMA..

[6] Arabi Y, Venkatesh S, Haddad S, Al Malik S, Al SA (2004). The characteristics of very short stay ICU admissions and implications for optimizing ICU resource utilization: the Saudi experience. Int J Qual Health Care..

[7] Zimmerman JE, Kramer AA (2010). A model for identifying patients who may not need intensive care unit admission. J Crit Care..

[8] Junker C, Zimmerman JE, Alzola C, Draper EA, Wagner DP (2002). A multicenter description of intermediate-care patients: comparison with ICU low-risk monitor patients. Chest..

[9] Prin M, Wunsch H (2014). The role of stepdown beds in hospital care. Am J Respir Crit Care Med..

[10] Rosenberg AL, Hofer TP, Hayward RA, Strachan C, Watts CM (2001). Who bounces back? Physiologic and other predictors of intensive care unit readmission. Crit Care Med..

[11] Franklin CM, Rackow EC, Mamdani B, Nightingale S, Burke G, Weil MH (1988). Decreases in mortality on a large urban medical service by facilitating access to critical care. An alternative to rationing. Arch Intern Med.

[12] Beck DH, McQuillan P, Smith GB (2002). Waiting for the break of dawn? The effects of discharge time, discharge TISS scores and discharge facility on hospital mortality after intensive care. Intensive Care Med..

[13] Campbell AJ, Cook JA, Adey G, Cuthbertson BH (2008). Predicting death and readmission after intensive care discharge. Br J Anaesth..

[14] Eachempati SR, Hydo LJ, Barie PS (2004). The effect of an intermediate care unit on the demographics and outcomes of a surgical intensive care unit population. Arch Surg..

[15] Ranhoff AH, Rozzini R, Sabatini T, Cassinadri A, Boffelli S, Ferri M (2006). Subintensive care unit for the elderly: a new model of care for critically ill frail elderly medical patients. Intern Emerg Med..

[16] Peelen L, de Keizer NF, Peek N, Scheffer GJ, van der Voort PH, de Jonge E (2007). The influence of volume and intensive care unit organization on hospital mortality in patients admitted with severe sepsis: a retrospective multicentre cohort study. Crit Care..

[17] Teli M, Morris-Stiff G, Rees JR, Woodsford PV, Lewis MH (2008). Vascular surgery, ICU and HDU: a 14-year observational study. Ann R Coll Surg Engl..

[18] Ranzani OT, Zampieri FG, Taniguchi LU, Forte DN, Azevedo LC, Park M (2014). The effects of discharge to an intermediate care unit after a critical illness: a 5-year cohort study. J Crit Care..

[19] Bellomo R, Goldsmith D, Uchino S, Buckmaster J, Hart G, Opdam H (2005). A before and after trial of the effect of a high-dependency unit on post-operative morbidity and mortality. Crit Care Resusc..

[20] Solberg BC, Dirksen CD, Nieman FH, van Merode G, Ramsay G, Roekaerts P (2014). Introducing an integrated intermediate care unit improves ICU utilization: a prospective intervention study. BMC Anesthesiol..

[21] Capuzzo M, Volta CA, Tassinati T, Moreno RP, Valentin A, Guidet B (2014). Hospital mortality of adults admitted to intensive care units in hospitals with and without intermediate care units: a multicentre European cohort study. Crit Care..

[22] Keenan SP, Massel D, Inman KJ, Sibbald WJ (1998). A systematic review of the cost-effectiveness of noncardiac transitional care units. Chest..

[23] Bertolini G, Confalonieri M, Rossi C, Rossi G, Simini B, Gorini M (2005). Costs of the COPD. Differences between intensive care unit and respiratory intermediate care unit. Respir Med.

[24] Solberg BC, Dirksen CD, Nieman FH, van Merode G, Poeze M, Ramsay G (2008). Changes in hospital costs after introducing an intermediate care unit: a comparative observational study. Crit Care..

[25] Byrick RJ, Mazer CD, Caskennette GM (1993). Closure of an intermediate care unit. Impact on critical care utilization. Chest..

[26] Kahn JM, Rubenfeld GD, Rohrbach J, Fuchs BD (2008). Cost savings attributable to reductions in intensive care unit length of stay for mechanically ventilated patients. Med Care..

[27] Chen LM, Kennedy EH, Sales A, Hofer TP (2013). Use of health IT for higher-value critical care. N Engl J Med..

[28] Cheng SH, Jan IS, Liu PC (2008). The soaring mechanic ventilator utilization under a universal health insurance in Taiwan. Health Policy..

[29] Kahn JM, Benson NM, Appleby D, Carson SS, Iwashyna TJ (2010). Long-term acute care hospital utilization after critical illness. JAMA..

[30] Zimmerman JE, Wagner DP, Knaus WA, Williams JF, Kolakowski D, Draper EA (1995). The use of risk predictions to identify candidates for intermediate care units. Implications for intensive care utilization and cost. Chest..

[31] Vincent JL, Burchardi H (1999). Do we need intermediate care units?. Intensive Care Med..

[32] Jacobs P, Rapoport J, Edbrooke D (2004). Economies of scale in British intensive care units and combined intensive care/high dependency units. Intensive Care Med..

[33] Kanhere MH, Kanhere HA, Cameron A, Maddern GJ (2012). Does patient volume affect clinical outcomes in adult intensive care units?. Intensive Care Med..

[34] Department of Health: Comprehensive critical care: a review of adult critical care services. National Archives. 2000. http://webarchive.nationalarchives.gov.uk/+/www.dh.gov.uk/en/publicationsandstatistics/publications/publicationspolicyandguidance/dh_4006585.

[35] Gershengorn HB, Iwashyna TJ, Cooke CR, Scales DC, Kahn JM, Wunsch H (2012). Variation in use of intensive care for adults with diabetic ketoacidosis. Crit Care Med..

